# Quality of Life and Costs in Parkinson's Disease: A Cross Sectional Study in Hungary

**DOI:** 10.1371/journal.pone.0107704

**Published:** 2014-09-17

**Authors:** Gertrúd Tamás, László Gulácsi, Dániel Bereczki, Petra Baji, Annamária Takáts, Valentin Brodszky, Márta Péntek

**Affiliations:** 1 Department of Neurology, Semmelweis University, Budapest, Hungary; 2 Department of Health Economics, Corvinus University of Budapest, Budapest, Hungary; 3 Rheumatology, Flór Ferenc County Hospital, Kistarcsa, Hungary; University of Toronto, Canada

## Abstract

**Background:**

Patient reported outcomes and costs of illness are useful to capture some of the multiple effects of a disease and its treatments. Our aim was to assess quality of life (QoL) and costs of Parkinson's disease (PD) in Hungary, and to analyze their associations.

**Methods:**

A cross-sectional questionnaire survey was conducted in one neurology university clinic. Clinical characteristics, PD related resource utilizations and productivity loss in the past 12 months were recorded; the Hoehn&Yahr (HY) scale, PDQ-39 and EQ-5D questionnaires were applied. Cost calculation was performed from the societal perspective.

**Results:**

110 patients (34.5% female) were involved with mean age of 63.3 (SD = 11.3) and disease duration of 8.2 (SD = 5.8) years. PDQ-39 summary score was 48.1 (SD = 13.4). The average EQ-5D score was 0.59 (SD = 0.28), and was significantly lower than the population norm in age-groups 45–74. The correlation was significant between EQ-5D and PDQ-39 (−0.47, p = 0.000), the HY scale and EQ-5D (−0.3416, p = 0.0008) and PDQ-39 (0.3419, p = 0.0006) scores. The total mean cost was €6030.2 (SD = 6163.0)/patient/year (direct medical 35.7%, direct non-medical 29.4%, indirect cost 34.9%). A one year increase in disease duration and 0.1 decrease of the EQ-5D utility score increase the yearly costs by 8 to 10%, and 7.8%, respectively. The effect of the PDQ-39 score on total cost was not significant.

**Conclusions:**

Disease severity and public health importance of PD are clearly demonstrated by the magnitude of QoL loss. PD-related costs are substantial, but are much lower in Hungary than in Western European countries. Disease duration and EQ-5D score are significant proxy of costs.

## Introduction

Parkinson's disease (PD) is one of the most common neurodegenerative disorders [1]. In Hungary, about 20 000 people live with PD [2]. Over the past two years, cost of illness studies from various European countries have been reported that PD related costs substantially vary from country to country [3]–[7]. In Central Eastern Europe detailed cost of illness analysis in PD was published only from the Czech Republic and Russia [6]–[7], therefore, estimation of the disease burden from PD in Europe is very limited. Local data and evidence in the country-specific context are needed for health economic evaluations that have become increasingly important in PD with the spread of advanced device-aided therapies.

Our aim was to provide evidence on health-related quality of life (HRQL) of patients with PD and cost of illness in Hungary, and to analyze the relationship between diverse health measures and costs.

## Methods

### Patients

Patients with established diagnosis of PD attending routine care between November 2008 and March 2010 at the Department of Neurology, Semmelweis University, in Budapest were invited to participate in a cross-sectional survey. Ethical approval (reference number: 10796-0/2010-1018EKU) was obtained from the Hungarian Medical Research Council. Patients signed informed consent form. All of the patients had the cognitive ability to consent according to the findings in the neurological examination; therefore, there was no surrogate consent procedure.

### Questionnaire

Demographic data, employment status, main clinical characteristics, PD related medication, use of health care services in the past 12 months, transportation utilizations to attend medical care, and help from others for everyday activities (informal care) were all surveyed.

Neurologists categorized patients by disease severity using the Hoehn&Yahr scale (HY) [8]. Patients' general health status was measured with the EQ-5D-3L (hereinafter EQ-5D) questionnaire and a Visual Analogue Scale (EQ VAS) [9]. Due to lack of national tariffs the UK algorithm was used to calculate the EQ-5D utility score. The health-related quality of life was assessed by the PDQ-39 [10].

### Costs

Hungarian official costs, tariffs and reimbursement lists of 2009 and the utilization data reported by patients were used for cost calculation. The cost of consultation was calculated by multiplying the number of visits by the estimated unit prices (GP: €5.5/visit, out-patient specialist: €6.7/visit). The costs of hospitalizations were based on Disease Related Groups reimbursement lists (€825.54 per occasion). Diagnostic procedures were evaluated on official outpatient prices (e.g. skull MRI: €56.6/case). Drug costs were calculated based on official prices of pharmaceuticals and the doses reported by the patient. We did not involve drugs treating non-motor symptoms to the analysis similarly to other cost estimating studies [6]–[7]. Non-reimbursed medical services' costs were based upon patients' answers. The cost of ambulance was calculated by the estimated costs/km (€3.7/km), multiplied by the distance of the patient's home to the clinic and the number of utilizations.

Transportation cost of patient and the accompanying person was calculated considering the way of transportation used and the distance between patients' residence and neurology care. Weekly cost of informal care was calculated using the average hourly net wage in Hungary (€2.8/hour) multiplied by the number of hours/week, and it was projected to a 12 months period but capped for the yearly net wage.

The costs of sick leave, part time job and full disability due to PD were calculated using human capital approach method. Average gross income (€957/month in 2009) including net wage, personal income tax, pension contribution, health insurance contributions, employers' contribution was multiplied with the time off work. Conversion (280.6 Hungarian Forints = €1) was applied.

### Statistical analysis

We used Student t-test to compare the EQ-5D scores of PD patients to the general Hungarian population by age groups, the significance level of 5% was applied. We also calculated pair-wise correlation between EQ-5D scores, EQ VAS, PDQ-39 indices and HY categories. We used one-way ANOVA test to examine, whether the costs significantly differ by HY categories. We examined the relation between health-related indices, disease severity and costs using multivariate linear regression analysis. We used the logarithm of the cost as dependent variable; age and gender of the patients, HRQL indices (EQ-5D, EQ VAS, PDQ-39) and disease severity (HY category) as independent variables. We built four models separately for the four independent variables. Stata 11 software was used for the analysis.

## Results

### Main characteristics and health status of the sample

Main characteristics of the patient sample (N = 110) are summarized in Table 1 and Table S1.

**Table 1:** Characteristics of patients with Parkinson's disease.

Variables	Mean (SD)
Age, year	(N = 110) 63.3 (11.3)
Disease duration, year	(N = 108) 8.2 (5.8)
Weight, kg	(N = 109) 74.1 (13.1)
Height, cm	(N = 108) 169.7 (8.9)
Body Mass Index	(N = 108) 25.6 (3.7)

	Number of patients (%)
**Gender**	
females	38 (34.5)
males	70 (63.6)
missing data	2 (1.8)
**Hoehn**&**Yahr scale**	
I	20 (18.2%)
II	30 (27.3%)
III	41 (37.3%)
IV	11 (10.0%)
V	0 (0.0%)
Missing data	8 (7.3%)

For details see also Table S1.

### EQ-5D

**Figure 1:**
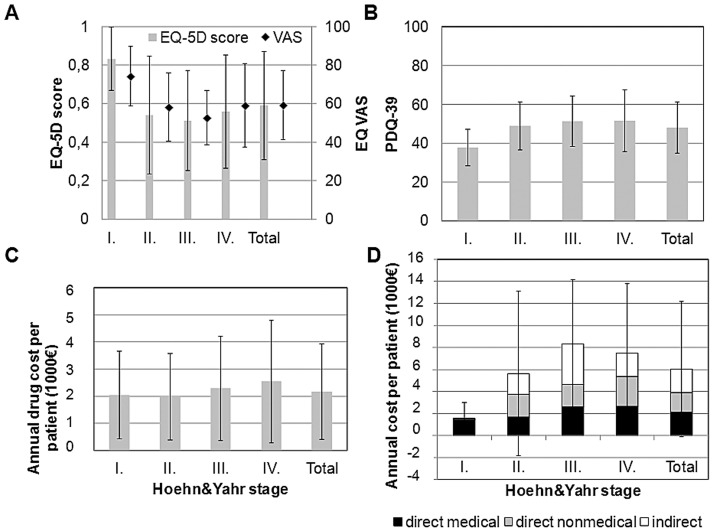
Quality of life, costs of medication, cost components in the Hoehn&Yahr categories. **A**: EQ-5D and VAS scores **B**: PDQ-39 index scores **C**: Costs of medication therapy related to Parkinson's disease **D**: Distribution of cost components. Vertical lines indicate standard deviations.

**Figure 2:**
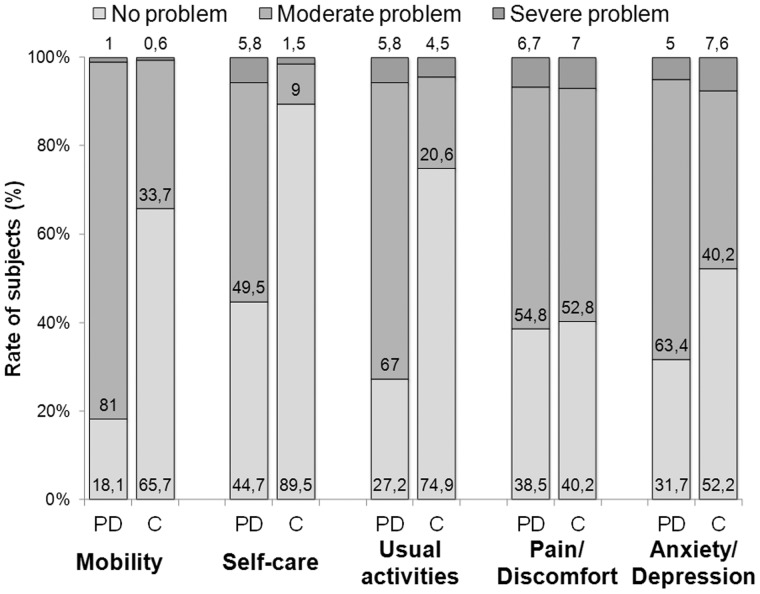
EQ-5D results in Parkinson's disease and the general population in Hungary. Rate of PD patients reporting different levels of problem in the EQ-5D scale, in comparison with the general population in Hungary [11]. PD: Patients with PD, age mean 63.3 (SD: 11.3) years, N = 110; C: control, general population, age-group 60–69 yrs, N = 721.

The EQ-5D and EQ VAS scores were mean 0.59 (SD = 0.28) and 59.3 (SD = 17.9), respectively (Figure 1A). Patients with PD had a significantly lower average EQ-5D score in age-groups 45–54 (N = 14), 55–64 (N = 37), 65–74 (N = 34) than the general population (p<0.05) [11]. The difference was not significant in age-group 75–84 (N = 16). The number of patients in age groups 25–34, 35–44 and ≥85 was not sufficient for the analysis (N = 1; 5 and 3, respectively). Mobility was the most problematic health area for the PD patients (Figure 2).

### PDQ-39

The average total score of PDQ-39 was 48.1 (median: 47.8; SD = 13.4; min-max: 8.3–79.4) (Figure 1B) and the mean scores by health dimensions were as follows: mobility: 50.0 (median: 47.5; SD = 21.4; min-max: 0–100), activities of daily living: 51.3 (median 50; SD = 22.1; min-max: 0–100), emotional well-being: 52.8 (median: 47.9; SD = 20.6; min-max: 0–100), stigma: 50.8 (median: 43.8; SD = 24.4; min-max: 0–100), social support: 32.5 (median: 25; SD = 15.0; min-max: 0–100), cognitions: 45.3 (median: 43.8; SD = 19.3; min-max: 12.5–100), communication: 43.8 (median: 47.1; SD = 18.8; min-max: 8.3–100), bodily discomfort: 58.2 (median: 58.3; SD = 21.9; min-max: 0–100).

### Health status by disease severity and correlation of HRQL scores

Results by HY categories are presented on Figure 1A,B. We found significant differences in EQ-5D, EQ VAS and PDQ-39 scores between the HY category I and II (p<0.01). However, the differences were not significant neither between the HY group II and III, nor between III and IV. We found a significant moderate correlation between the EQ-5D and EQ VAS scores (0.49; p = 0.000) as well as between EQ-5D and PDQ-39 scores (−0.47; p = 0.000). Significant but weaker correlation was found between EQ VAS and PDQ-39 (−0.31; p = 0.002), as well as between the HY scale and the EQ-5D (−0.3416, p = 0.0008), the EQ VAS (−0.3683, p = 0.0004) and the PDQ-39 score (0.3419, p = 0.0006). The correlation between disease duration and EQ-5D score was significant but moderate (−0.32, p = 0.0015), and significant but week between disease duration and PDQ-39 score (0.25, p = 0.0117).

We found a moderate significant correlation between disease duration and direct medical costs (0.40, p = 0.000) as well as low but significant correlation between disease duration and total costs (0.25, p = 0.0095). The correlation was not significant between disease duration and direct non-medical costs, nor it was between disease duration and indirect costs (p>0.05).

### Health care utilization and costs

One patient did not provide data on healthcare utilization therefore cost of illness calculation was performed considering a sample of 109 patients. At the time of the survey 94 (86.2%) patients were on medication due to PD. None of the patients had surgery in the last 12 months.

The average yearly total cost per patient was €6030.2 (SD = 6163.0), distribution between direct medical, direct non-medical and indirect cost was 35.7%, 29.4% and 34.9%, respectively (Table 2). Drugs contributed to 75% of the direct medical costs (Table 2 and Table S2), and drug cost increased with disease severity (Figure 1C).

**Table 2:** Resource utilization and costs of patients with Parkinson's disease in Hungary.

	Resource utilisation		Average yearly cost, EUR/patient/year	
	Rate of patients with at least one occasion (%)	Mean of the quantity used in the past 12 months (n = 109)	Cost per patient (total sample)	Cost category (total sample)
**Consultations (outpatient)**	-	-	**50.9**	**Direct medical cost: 2149.3 Euro/patient/year (35.7%)**
GP visit	68 (62.3%)	3.3	17.8	
Specialist visit	100 (91.7%)	4.9	33.1	
**Hospital admission**	37 (33.9%)	0.4	**348.4**	
**Diagnostics**	-	-	**42.5**	
CT scan	68 (62.4%)	0.4	8.0	
MRI scan	54 (49.5%)	0.2	11.4	
SPECT	17 (15.6%)	0.04	17.8	
Laboratory tests	67 (601.5%)	0.8	0.7	
X-ray	28 (25.7%)	0.1	0.5	
Neuropsychology	41 (37.6%)	0.3	3.4	
Doppler	21 (19.3%)	0.1	0.4	
Tremor analysis	18 (16.5%)	0.1	0.2	
Tests of autonomic functions	21 (19.3%)	0.2	0,1	
**Drugs (present users)**	-	-	**1614.3**	
MAO-B inhibitor	35 (32.1%)		179.1	
Amantadine	29 (26.6%)		33.5	
Anticholinergic drug	4 (3.7%)		3.9	
Dopamin agonist	50 (45.9%)		484.7	
Levodopa+decarboxylase inhibitor	59 (54.1%)		117.9	
Levodopa+decarboxylase inhibitor+COMT inhibitor	34 (31.2%)	-	535.0	
COMT-inhibitor	18 (16.5%)	-	260.2	
**Other health care services**	-	-	**93.2**	
Not reimbursed services	19 (17.4%)	-	57.2	
Ambulance	8 (7.3%)	0.2	36.0	
**Non-medical cares**	**-**	**-**	**1774.6**	**Direct non-medical cost: 1774.6 EUR (29.4%)**
Transportation	92 (84.45%)	-	69.9	
Informal care	47 (43.1%)	12.6 hours/week	1704.7	
**Productivity loss**	**-**	**-**	**2106.3**	**Indirect cost: 2106.3 EUR (34.9%)**
Work disability pension*	19 (17.4%)	NA	2002.7	
Sick leave	5 (4.6%)	1.6 days/year	75.1	
Part time job due to PD	1 (0.9%)	NA	28.5	
**Total**	NA	NA	NA	**6030.2 EUR**

For cost of drugs see also Table S2.

**(N = 109; conversion €1 = 280.6 HUF);**

* All of them are on disability pension due to their Parkinson's disease.

The annual average cost per patient by HY categories were I: €1543.3 (SD = 1451.4), II: €5620.9 (SD = 7478.2), III: €8325.5 (SD = 5811.4) and IV: €7462.3 (SD = 6302.2), respectively, and none of the patients were in HY-V stage (Figure 1D). According to the results of the ANOVA test, total cost was significantly influenced by HY stages (F_3,97_ = 6.22, p = 0.0007). Post-estimations revealed significant difference between the cost in HY-I and II stages (F_1,97_ = 5.69 p = 0.0190), but not in HY-II and III (F_1,97_ = 3.59, p = 0.0610), and in HY-III and IV stages (F_1,97_ = 0.19, p = 0.6665).

We found significant differences between HY stages in direct medical costs (F_3,97_ = 4.86, p = 0.0034), namely between HY stages II and III (F_1,97_ = 7.50, p = 0.0074), as well as in indirect costs (F_3,97_ = 3.24, p = 0.0254), namely between stages II and III (F_1,97_ = 2.93, p = 0.0900). At the same time, we did not find significant differences between HY stages in direct nonmedical costs (F_3,97_ = 1.90, p = 0.1348).

### Regression analysis

Results are presented in Table 3. The first three models built for the logarithm of total costs with different independent variables for the EQ-5D, EQ VAS and PDQ-39 separately, are significant at 5% levels (p<0.05). We found that one year progression of the disease increases the yearly costs by 8.0%, 9.9% and 9.3%, depending on the model specification. A 0.1 point decrease of the EQ-5D score results in 7.8% increase of the cost while 1 point decrease on the EQ VAS scale causes 2.3% cost increase. The relationship between the PDQ-39 and total cost was not significant.

**Table 3:** Results of the regression analysis models built for the EQ-5D, EQ VAS and PDQ-39 separately.

Variables	Model 1 – EQ-5D	Model 2 – EQ VAS	Model 3 – PDQ-39	Model 4 – HY†
	ln (total cost)	ln (total cost)	ln (total cost)	ln (total cost)
Age	−0.021 (0.013)	−0.027** (0.014)	−0.0168 (0.013)	−0.0338*** (0.012)
Male	−0.325 (0.317)	−0.356 (0.328)	−0.274 (0.311)	−0.466* (0.277)
Disease duration	0.076*** (0.028)	0.094*** (0.027)	0.0885*** (0.027)	0.0194 (0.032)
EQ-5D	−1.530*** (0.561)	-	-	-
EQ VAS	-	−0.023** (0.008)	-	-
PDQ-39	-	-	0.0142 (0.011)	-
HY I	-	-	-	−2.457*** (0.450)
HY II	-	-	-	−0.366* (0.189)
HY IV	-	-	-	−0.112 (0.115)
Constant	15.42*** (0.977)	16.14*** (1.162)	13.44*** (0.971)	16.60*** (0.899)
Observations	95	91	101	99
F	5.81	5.89	4.10	9.21
P	0.0003	0.0003	0.0041	0.000
R-squared	0.205	0.215	0.146	0.375

*p<0.1,

** p<0.05,

*** p<0.01;

† HY III was used as reference category in Model 4; t-statistics in parentheses.

In the fourth model, when we include the severity categories (HY) as dummy variables controlled for age, gender and disease duration, we find that compared to the HY III group, the costs of the HY I and HY II groups are significantly lower, by 91.3% and 30.7%, respectively. The difference between average annual cost per patient between groups HY IV and III was not significant. In this model age and gender are also significant, while disease duration is not.

## Discussion

In this study, we have assessed HRQL and costs of illness of PD in a cross-sectional questionnaire survey involving 110 patients and compared different health measures and costs.

The PDQ-39 questionnaire revealed the highest deterioration in the bodily discomfort and emotional well-being areas, however similar impairment was detected in mobility, activities of daily living and stigma as well. The average PDQ-39 summary index score in Hungary (48.1, SD = 13.4) was comparable to the findings in Spain [12] (48.8, SD = 27.8) and Croatia [13] (median SI: 47, interquartile range: 32–62) but was higher than in Germany [14] (29.4, SD = 17.5), France [15] (36.5, SD = 14.2), Norway [16] (18.7, SD = 12.0) and Poland [17] (32.17, SD = 16.82) at a similar mean age and disease duration. It thus seems that disease-specific HRQL of PD patients are worse in Hungary than in economically more developed European countries. It can be inferred that the differences originate from various factors, including social support, co-morbidities and access to chronic care [18].

The EQ-5D score was lower in PD than in the age matched general population in Hungary; the most affected health dimensions were mobility and the performance of usual activities. In a study across 10 countries, Martinez-Martin et al. [19] reported similar average EQ-5D scores at a similar mean age (64.5, SD = 9.9) and disease duration (8.1, SD = 5.7).

We found significant moderate correlation between the PDQ-39 summary score and the EQ-5D utility index, but the correlation with the EQ VAS was weaker, which is in line with international findings [20]. The correlation was significant, but moderate between HY stages and the three health indices. Our results confirm the limited capacity of HY medical categorical scale to consider important HRQL aspects, thus HY based cost-effectiveness models using EQ-5D utility values should be evaluated in appropriate sensitivity analyses [21].

The cost of illness of PD was mean €6030/patient/year in Hungary (Table 2). Among drugs, the combination of levodopa, decarboxylase inhibitor and COMT-inhibitor (Stalevo) was of the highest cost, followed by dopamine agonists. Subcutan apomorfin pump is not yet available in Hungary, and patients with deep brain stimulation or jejunal Levodopa/carbidopa infusion were not in the sample, so their contributions to PD-related costs in Hungary are still lacking.

Informal care provided by family members, or volunteers occurred in 43% of the patients, which contributed to 43% of the direct costs (Table 2). It seems that in Hungary, PD related disability mainly burdens the patients' families, and not the health and social care system. Despite the relatively high average age of the sample unemployment affected about one quarter of the patients, thus indirect costs were the highest cost category (35%). It is worthy to note that the standard deviation (SD) of costs was quite large (mean 6030.2, SD = 6163.0 euros/patient/year). Although it was observed in all the three cost categories, the largest SD was found for indirect cost category. For those patients who were working or were retired the productivity lost was 0, while for those who were disabled due to their PD we calculated with a one year gross wage loss which was 11,5 thousand euros in Hungary. This partly explains the high standard deviation of annual cost.

One major conclusion of a comparative study in six countries was that total costs from a societal perspective were lower in Eastern, than in Western European countries. Our results seem to strengthen this observation, although direct comparison is hampered by methodological differences. The total costs of PD in Hungary is expressed in purchasing power parity (PPP), 2009 euros was 10 144 €/patient/12 months (conversion PPP 2009 €1 = 166.8 HUF), which is closer to the Czech result (PPP 2008 €5510/patient/6 months), than to Austria, Germany, or Italy (9820, 8610 and 8340 PPP 2008 €/patient/6 months, respectively) [6]. In Hungary the medication costs were the main drivers of direct costs. Similar to other European countries, and Russia [6], the levodopa+decarboxylase inhibitor, or its combination with COMT-inhibitor and dopamine-agonists, were most often prescribed in Hungary, but MAO-B inhibitors were more frequently used in our sample (Table 2 and Table S2), than most of these countries except Portugal. Hospitalisation amounted to only 9% (€348/patient/year) in Hungary, whilst it has been described as one of the major cost factors in Western European countries, comprising of 13%–42% of total direct costs. Informal care in PD has been analyzed in five publications up to date, and its unit cost varied between 2.36–7.99 USD (2010), and the average time from 8.90 to 17.25 hours/week [22]. It seems that informal care usage in Hungary is in the upper range (12.6 hours/week), but its unit cost is in the lower band (€2.8/hour).

Analysis by HY categories revealed a significant increase of total cost between stages I and II, but not between II and III, and stages III and IV in Hungary (Figure 1D). Drug costs (Figure 1C), and direct medical costs increase with HY stages, but indirect costs decrease between HY III–IV because all patients with the exception of two disability pensioners were retired in the HY IV subgroup. Thus, indirect costs were much lower in HY IV state, as out of the 11 patients in the HY IV subgroup 9 were retired with no productivity loss considered in their case. von Campenhausen et al. found a similar trend in Portugal, however, in other countries the increase of total costs was observed between HY III–IV [6]. Disease duration significantly correlated with direct medical costs and total costs, but not with direct non-medical costs, and indirect costs in our study.

In our study regression analysis confirmed significant relationship between EQ-5D score and total costs. A 0.1 decrease of the EQ-5D utility score increases the yearly costs by 7.8%, and one year increase in disease duration raises it by 8–10% (Table 3). Further studies, including more patients in worse HY stages, and considering other clinical factors are required to confirm and refine our observations.

The average yearly cost per patient in multiple sclerosis [23] and schizophrenia [24] is about 1.7 and 2.3 higher compared to PD in Hungary and costs of dementia [25] is also slightly higher. A model-based estimate found similar trends in Europe; however, they estimated a lower average cost for PD in Hungary on the national level (PPP 2010 €5926) using extrapolations [26].

Limitations of our study have to be considered for the interpretation of the results. The survey was performed in one neurology university clinic, thus results cannot be generalized to the whole PD patient population in Hungary. Patients in the most severe health state (HY V category) were not involved. Regarding informal care, we considered only caregivers' time based on patients' answers and did not collect further data on caregivers' employment status, productivity and quality of life loss. Nonetheless, there is a large methodological heterogeneity in the cost of illness literature. We strongly agree with Dodel et al. [21] that ‘there is a need for a validated instrument that would provide more reliable and transparent cost data for patients with PD to estimate better the burden of the disease’. Taking into consideration the significant rate of informal care in PD, this would be especially important for the assessment and costing of help provided by others for everyday life. Further studies, involving more representative samples might confirm or refine our results. Nevertheless our study contributes with important observations to health economic evaluations, and a better understanding of disease burdens of PD across different countries.

## Supporting Information

Table S1 **Detailed characteristics of the patients.**
(DOCX)

Table S2 **Details for drug utilization and costs of patients.**
(DOCX)
