# Supplementary material for: Quality of Life and Costs in Parkinson's Disease: A Cross Sectional Study in Hungary
Source: PLoS One. 2014 Sep 17;9(9):e107704. doi: 10.1371/journal.pone.0107704 (PMC4167855; doi:10.1371/journal.pone.0107704)
Supplement: Table S1 — Detailed characteristics of the patients. (DOCX) [file pone.0107704.s001.docx]

**Table S1.**

| **Variables** | **Number of patients (%)** |
| --- | --- |
| **Age, years** |  |
| 25-34 | 1 (0.9%) |
| 35-44 | 6 (5.5%) |
| 45- 54 | 14 (12.7%) |
| 55- 64 | 37 (33.6%) |
| 65- 74 | 35 (31.8%) |
| 75 - | 17 (15.5%) |
| **Body Mass Index (BMI)** |  |
| <18.5 (underweight) | 7 (6.4%) |
| 18.5-24.9 (normal) | 42 (38.2%) |
| ≥25 (overweight or obese) | 59 (53.6%) |
| Missing data | 2 (1.8%) |
| **Marital status** |  |
| Single | 4 (3.6%) |
| Married or living together | 83 (75.5%) |
| Divorced | 9 (8.2%) |
| Widow | 10 (9.1%) |
| Missing data | 4 (3.6%) |
| **Highest educational level** |  |
| Primary school | 21 (19.1%) |
| Secondary school | 44 (40.0%) |
| Bachelor degree | 16 (14.5%) |
| Master degree | 24 (21.8%) |
| Missing data | 5 (4.5%) |
| **Employment status** |  |
| Works in a full time job | 16 (14.6%) |
| Works in part-time job | 1 (0.9%) |
| Pensioner, working full time | 1 (0.9%) |
| Pensioner, working part time | 1 (0.9%) |
| Disability pensioner (< age 62), working part time | 1 (0.9%) |
| Disability pensioner* (< age 62) | 19 (17.3%) |
| Retired (age **≥** 62) | 62 (56.4%) |
| Unemployed | 2 (1.8%) |
| Other | 2 (1.8%) |
| Missing data | 5 (4.5%) |
| **Net income, €/month**  **conversion €1=280.6 HUF** |  |
| 0-255 | 21 (19.1%) |
| 256- 410 | 38 (34.5%) |
| 411 – 569 | 28 (25.5%) |
| 570 – 926 | 12 (10.9%) |
| 927 – 2315 | 1 (0.9%) |
| 2316 - | 0 (0.0%) |
| Missing data | 10 (9.1%) |

***^All of them are on disability pension due to their Parkinson’s disease^**
