# Supplementary material for: Quality of Life and Costs in Parkinson's Disease: A Cross Sectional Study in Hungary
Source: PLoS One. 2014 Sep 17;9(9):e107704. doi: 10.1371/journal.pone.0107704 (PMC4167855; doi:10.1371/journal.pone.0107704)
Supplement: Table S2 — Details for drug utilization and costs of patients. (DOCX) [file pone.0107704.s002.docx]

**Table S2.**

|  | **Resource utilisation** | **Cost** |
| --- | --- | --- |
|  | Rate of patients with at least one occasion (%) | Average annual cost, EUR/patient/year (total sample) |
| **Drugs (present users)** | - | **1614.3** |
| MAO-B inhibitor | 35 (32.1%) | 179.1 |
| (Azilect) | *15 (13.8%)* | *158.5* |
| (Jumex),  (Selegiline Merck),  (Selegiline-Chinoin) | *21 (19.3%)* | *20.6* |
| Amantadine | 29 (26.6%) | 33.5 |
| (PK-Merz) | *23 (21.1%)* | *28.9* |
| (Viregyt-K) | *6 (5.5%)* | *4.6* |
| Anticholinergic drug | 4 (3.7%) | 3.9 |
| (Akineton) | *3 (2.8%)* | *3.3* |
| (Tremaril) | *1 (0.9%)* | *0.6* |
| Dopamin agonist | 50 (45.9%) | 484.7 |
| (Requip) | *9 (8.3%)* | *35.6* |
| (Requip-Modutab) | *37 (34.0%)* | *279.1* |
| (Mirapexin) | *12 (11.0%)* | *170.0* |
| Levodopa+decarboxylase inhibitor | 59 (54.1%) | 117.9 |
| (Sinemet CR) | *9 (8.3%)* | *12.3* |
| (Madopar) | *41 (37.6%)* | *76.5* |
| (Madopar HBS) | *5 (4.6%)* | *5.7* |
| Solubile (Madopar) | *15 (13.8%)* | *23.4* |
| Levodopa+decarboxylase inhibitor+COMT inhibitor: (Stalevo) | 34 (31.2%) | 535.0 |
| COMT-inhibitor: (Comtan) | 18 (16.5%) | 260.2 |
